# Through the lens of phase separation: intrinsically unstructured protein and chromatin looping

**DOI:** 10.1080/19491034.2023.2179766

**Published:** 2023-02-23

**Authors:** Ling Cai, Gang Greg Wang

**Affiliations:** a Lineberger Comprehensive Cancer Center, University of North Carolina at Chapel Hill School of Medicine, Chapel Hill, NC, USA; b Department of Genetics, University of North Carolina at Chapel Hill School of Medicine, Chapel Hill, NC, USA; c Department of Biochemistry and Biophysics, University of North Carolina at Chapel Hill School of Medicine, Chapel Hill, NC, USA; d Department of Pharmacology, University of North Carolina at Chapel Hill School of Medicine, Chapel Hill, NC, USA

**Keywords:** Phase separation, three-dimensional chromatin structure, intrinsically disordered proteins (IDPs), intrinsically disordered regions (IDRs), DNA looping, CTCF, cohesin, enhancer-promoter interaction, loop extrusion, cancer

## Abstract

The establishment, maintenance and dynamic regulation of three-dimensional (3D) chromatin structures provide an important means for partitioning of genome into functionally distinctive domains, which helps to define specialized gene expression programs associated with developmental stages and cell types. Increasing evidence supports critical roles for intrinsically disordered regions (IDRs) harbored within transcription factors (TFs) and chromatin-modulatory proteins in inducing phase separation, a phenomenon of forming membrane-less condensates through partitioning of biomolecules. Such a process is also critically involved in the establishment of high-order chromatin structures and looping. IDR- and phase separation-driven 3D genome (re)organization often goes wrong in disease such as cancer. This review discusses about recent advances in understanding how phase separation of intrinsically disordered proteins (IDPs) modulates chromatin looping and gene expression.

## Introduction

Development of next-generation sequencing technologies, especially those based on high-throughput chromosome conformation capture (Hi-C) [1–4] and derivatives such as micrococcal nuclease chromosome conformation assay (Micro-C) [5,6] and Capture Hi-C [7,8], as well as super-resolution fluorescence microscopy techniques [9], has allowed an unprecedent view into spatiotemporal organization of three-dimensional (3D) chromatin structures during organismal development and cell differentiation [10–19]. Appropriate folding and spatial partitioning of 3D genome were proposed to be crucial for ensuring and/or facilitating a range of DNA-templated biological processes, such as coordinated co-transcription or co-silencing of genes within the same compartments (such as euchromatin and heterochromatin), the orderly genome replication, and genome integrity [11,13–21]. On the other hand, misregulation of 3D chromatin structure has been widely linked to, sometimes found to be causal for, development of diseases [22], including cancer [23–27], immunological malfunction [28], and neurological or developmental syndrome [29,30].

3D chromatin structure and genome folding are organized at different scales of genomic length [1,15,19–21,31–35], which at least include (i) segregation of large mega-base (Mb)-long regions into active (type A) and inactive (type B) compartments [1]; (ii) formation of sub-Mb domains termed Topologically Associating Domains (TADs), which have a median size of approximately 880 kilobase (kb) in mouse embryonic stem cells (ESCs) [3]; (iii) smaller compartmental domains, sometimes referred to as nested TADs or sub-TADs, that span over a size of a few to dozens of kb region and can cover one to several genes [4,17,19,31,35]; and (iv) chromatin loops such as long-range enhancer–promoter interactions [34–36]. Chromosomal regions that fall into the same compartment type (type A or B) interact with each other more frequently than those that do not. Likewise, TAD/sub-TAD represents a neighboring genomic region that shows a higher frequency of self-interaction than an equidistant region. Usually, TAD is enclosed by a chromatin loop, with its boundaries anchored by CCCTC-binding factor (CTCF) and cohesin, a ring-shaped multi-subunit complex that can entrap DNA inside its lumen [20,21,37].

**Figure 1.**
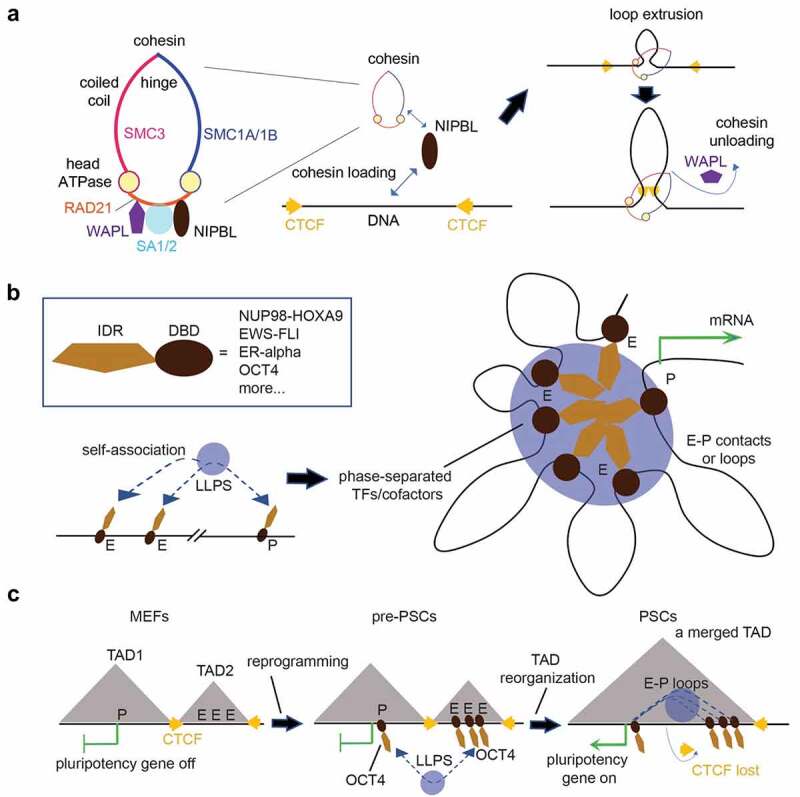
Chromatin looping driven by loop extrusion and phase separation. (a) A loop extrusion model to explain the generation of CTCF-/cohesin-associated structural loops/TADs. Left panel: composition and architecture of cohesin, which contains a core complex (SMC1-SMC3-RAD21) and the HEAT-repeat-containing regulatory factors such as SA1/SA2, NIPBL (a loader and activator of cohesin), and WAPL (a cohesin-releasing factor). Right panels: cohesin extrudes DNA and generates an enlarged loop until it is stalled by a pair of CTCF proteins in a convergent orientation. NIPBL loads cohesin to chromatin, whereas WAPL releases it off chromatin. (b) Enhancer (E)–promoter (P) looping due to transcription factor (TF) phase separation and condensation. Box: a simple, generalized scheme of TFs listed on the right, all of which harbor an intrinsically disordered region (IDR) and a DNA-binding domain (DBD). Long-range E–P interaction is potentiated by a phase separation mechanism, which involves self-association of TF IDRs and/or IDRs within coactivators (not shown). TF binding to E/P sites (left) and E–P looping (right) are the two highly coordinated processes that occur in condensates or the ‘hub’. Numerous cofactors (including protein and RNA; not shown) can be directly involved in condensate formation or passively recruited into condensates in a fashion consistent with the scaffold-client model. (c) OCT4-associated E-P loops cause TAD reorganization and activate pluripotency genes in an IDR-/LLPS-dependent manner. In mouse embryonic fibroblasts (MEFs), pluripotency genes are turned off partly due to CTCF structural TADs/loops that constrain E-P communication (left). During induced reprogramming of MEFs to pluripotent stem cells (PSCs), OCT4 binds E/P sites of pluripotency genes and starts to establish E–P loop clusters in a LLPS-dependent manner (middle and also see **B**). As a result, CTCF binding at TAD boundaries is decreased and adjacent TADs merge, leading to strong E–P contacts and pluripotency gene expression in PSCs (right).

Loop extrusion is a widely recognized model to explain the establishment of CTCF/cohesin loops and TADs [20,38–43]. Here, cohesin (Figure 1a, left) comprises a V-shaped dimer of Structural Maintenance of Chromosomes (SMC) family of ATPases (namely, SMC1A/1B and SMC3, all of which contain a ‘head’ domain carrying ATPase activity, a ‘hinge’ domain for dimerization of SMC1 and SMC3, and a long anti-parallel coiled-coil region connecting ‘head’ and ‘hinge’), RAD21, and one of two HEAT-repeat-containing subunits (namely, SA1 encoded by STAG1 or SA2 encoded by STAG2). Other HEAT-repeat-containing proteins, such as PDS5A/5B, NIPBL, and WAPL, also form contact with the core cohesin complex (SMC1-SMC3-RAD21) via interaction with RAD21 [20,21,44]. In the loop extrusion model, cohesin utilizes the ATP and ATPase-generated force for extruding DNA, which leads to progressive production of an enlarged loop (which can be ~Mb in size) until cohesin is stopped by a pair of CTCF proteins showing a convergent orientation [20,38–43] (Figure 1a, right). Cohesin is also regulated by loader/activator (Nipped-B-like [NIPBL] and MAU2 [45,46]) and a cohesin-releasing factor, WAPL **[**47,48**]**) (Figure 1a). The *WAPL-*deficient interphase cells exhibit a characteristic thread-like cohesin distribution, referred to as ‘vermicelli’ (a special pasta in Italian), and an unusual granular DNA staining pattern [47,48]; on the other hand, *Nipbl* depletion leads to genome-wide disappearance of TADs [32–34], resembling what was observed upon deletion of RAD21, an extruding motor core subunit [33,34].

Besides loop extrusion, other mechanisms and molecular forces are also critically involved in 3D genome folding [15,16,18–20,49]. Indeed, cohesin deletion causes a global loss of TADs and cohesin loops but does not affect overall patterning of the type A–type B compartmentalization; additionally, a subset of compartments even exhibited stronger interactions in cohesin-deficient cells than wild-type (WT) controls, suggesting that TAD formation and compartmentalization represent two independent processes [32–34]. As a matter of fact, compartments are more strongly correlated with their respective chromatin modification features in cohesin-deficient cells compared to WT (such as enhanced compartmentalization of type-A regions with histone acetylation, H3K4me3, and DNA accessibility or enhanced long-range interactions of polycomb-associated targets) [32–34]. Mechanisms other than loop extrusion mediate compartmentalization of chromatin.

Recently, the increasing amount of evidence points to critical roles of phase separation in determining and/or shaping the landscape of 3D chromatin structures [15,19,23,50–57]. In the next section, we will briefly cover the concept of phase separation and its involvements in establishing both higher-order (such as compartmentalization) and fine-scale 3D structures (such as loops). Then, the third section will be focused on recent advances in understanding the phase separation-based chromatin looping events, especially those driven by intrinsically disordered proteins (IDPs). IDPs refer to proteins enriched in intrinsically disordered regions (IDRs), which exhibit a strong tendency to phase separate [58–62]. As IDPs/IDRs are frequently associated with pathogenesis [23,63], an improved understanding of IDP-/IDR-associated 3D genome folding may help to develop new therapeutics for the affected patients.

## Phase separation of biomolecules and regulation of 3D chromatin structure

Phase separation refers to a type of weak, multivalent, and self-associating interaction that leads to the partitioning and condensation of biomolecules such as protein, DNA, and RNA and their complexes, without involvement of a sub-cellular membrane [55,57,64–80]. Phase separation, such as liquid–liquid phase separation (LLPS), is known to be involved in forming membraneless nuclear structures such as nucleoli, nuclear speckle, and Cajal body. In general, phase separation can be driven by multivalent ionic or hydrophobic interactions, which at least include hydrophobic stacking and pi–pi, electrostatic, and pi–cation interactions. For the detailed concept of phase separation and the underlying principles, the authors shall refer to recent comprehensive reviews [55,57,64–80]. Phase separation can also be established at different molecular scales, which range from relatively simple IDRs such as aromatic-residue-rich or charged-residue-rich sequences (some examples are those within FUS, a RNA-binding protein [81], and phenylalanine-glycine [FG] repeats in NUP98, a nucleoporin protein [82,83]) to repeating modular domains (such as SH3 domain repeats and their binding ligands in the nephrin–NCK–N-WASP system [79]) and to larger biomolecular polymers and interactors (for example, an array of modified mono-nucleosomes and readers such as BRD4 [80] and HP1-alpha [84,85], which are specifically associated with euchromatin and heterochromatin, respectively). Accordingly, phase separation is most likely to elicit very broad effects on 3D genome folding and looping, due to a wide range of molecular and genomic scales that it can operate on.

First, phase separation has been proposed to be a driving force of compartmentalization [15,19,23,50,51]. In the *in vitro* setting, Gibson et al. elegantly demonstrated LLPS of chromatin, a process modulated by various factors such as linker DNA, linker histone, and post-translational modifications [80]. In addition, BRD4, a dual-bromodomain-containing reader of histone acetylation co-mixes with nucleosomal arrays carrying its ligand (histone acetylation) to form liquid ‘droplets’ or condensates [80]. The observations of this work [80] and others [84–88] support a notion that LLPS enables the establishment, partitioning, and/or maintenance of compartments, exhibiting distinct chromatin modification features and transcriptional states. Misregulation of such a process is also relevant in human diseases. An example is BRD4-NUT, a chimeric fusion characterizing the NUT midline carcinoma (a rare but highly lethal cancer) [89,90]. BRD4-NUT forms nuclear condensates together with coactivators (such as p300) in a fashion consistent with LLPS, establishing the so-called megadomains that are abundantly enriched with histone acetylation and span over long genomic regions (up to 2 Mb in size) [91–94]; in addition, a Hi-C-based study demonstrated that these BRD4-NUT-associated megadomains, located on either the same or different chromosomes, interact with each other and form a spatially confined compartment termed ‘sub-compartment M’ [95]. More recently, another study of dividing cells showed a requirement of global histone deacetylation and subsequent phase transition of mitotic chromatin for proper segregation of chromosomes, which highlights a role for phase separation in mediating mitosis [96].

Loop extrusion has been an elegant and widely accepted model to explain key aspects regarding the generation of CTCF/cohesin loops and TADs; however, it does not specifically explain why merely a minor fraction of CTCF/cohesin-bound sites form loops [4,20]. Additional molecular determinants exist to regulate loop extrusion. Both cohesin and CTCF can phase separate *in vitro* and form the clustering or puncta patterns in the nucleus [97–100]. Hansen et al. studied an internal RNA-binding region (RBRi) of CTCF and found that RBRi not only directly interacts with RNA but is also required for CTCF self-association and clustering [98]. Comparison of murine ESCs carrying a RBRi-deleted CTCF mutant with WT controls showed that approximately 50% of all CTCF loops were lost (thus defined as RBRi-dependent CTCF loops), whereas type A and B compartments were not affected [98]. RBRi-deleted CTCF retains interaction with cohesin in the co-immunoprecipitation experiment, and a part of RBRi-dependent CTCF loops show no significant alterations in CTCF/cohesin binding at loop anchors [98]. Thus, loss of these loops in the mutant cells might be due to defects in CTCF-RNA binding, CTCF clustering, or both, since CTCF clustering and RNA binding are the two coordinated, RBRi-dependent processes. A model proposed that RNA-based self-association/clustering of CTCF makes it a more efficient boundary to cohesin-mediated extrusion [98]. In another study, Ryu et al. demonstrated phase separation of cohesin holocomplex to be dependent on the presence of long DNA (exceeding ~3kb), which also occurs independent of cohesin ATPase activity [97]. Cohesin/DNA condensation may modulate loop extrusion by forming a more stabilized structure at loop anchors, which awaits further investigation [97]. Furthermore, a recent study performed in human ESCs suggested a role of phase separation in ensuring insulation at about 20% of TAD boundaries [101]. These TAD boundaries generally display a lack of CTCF anchors, a high rate of transcription and spatial clustering, and an enrichment in housekeeping genes common to diverse cell types [101]. These observations together indicate that multivalent interaction and condensation of biomolecules (including classic loop extruding proteins and other factors) may modulate the loop extrusion process and shape the landscape of TADs and boundaries.

Transcription factors (TFs), chromatin modifiers, and cofactors often harbor IDRs, which can induce phase separation [58–62]. A model has been proposed that condensation and LLPS of transcriptional (co)regulator serve as a critical mechanism for forming and/or enhancing long-range chromatin looping and contacts [50,55–57]. In the next section, we will further discuss about recent advances in this area.

Finally, RNA represents another prominent class of contributing factors for 3D genome organization. RNA molecules such as those with N(6)-methyladenosine (m6A) modification [102]; a wide variety of RNA-binding, processing, and splicing factors [81,103–107]; and m6A readers [108] can undergo phase separation, which are not discussed in this focused review, and readers shall refer to other articles [71,104,109–112].

## IDP-/IDR-dependent and phase separation-driven chromatin looping

Recently, in-depth Hi-C/Micro-C mapping studies have demonstrated the existence of diverse classes of chromatin loops, which at least include cohesin/CTCF loops, long-range promoter–enhancer (E–P) loops, promoter–promoter (P–P) loops, and polycomb-associated contacts [32–36,113]. Although loop calling often relies on data quality, sequencing depth, and calling algorithm, Micro-C appears to be an approach more suitable for mapping fine-scale 3D structures such as E–P and P–P loops [35,36]—as shown in a recent study of mouse ESCs, many more of fine loops (~20,000 E–P loops and ~7,000 P–P loops) were called out, with a median size of ~100 kb [36]. As pointed out by Hsieh et al. [36], loops really refer to focal enrichments in contact frequency between a pair of genomic loci based on Hi-C/Micro-C maps; except those extrusion-driven cohesin/CTCF loops, the E–P, P–P, and polycomb-associated loops shall be more appropriately termed as interactions or contacts, as they may occur without actual looping [35,36]. Here, loop is a more freely used term to echo a textbook model of E–P looping [114], in a hope to make this review accessible to the general audience.

Distal regulatory elements such as enhancers play crucial roles in controlling gene transcription in a tissue- and cell type-specific manner. How exactly distal enhancers contribute to target gene expression has been a topic of intense investigation. In the classic E–P looping model, a tethering factor transiently brings promoter and enhancer into close proximity, thereby enhancing frequency of contacts between the two [114]. Recent studies have demonstrated that E–P loops/contacts and cohesin/CTCF loops exhibit quite diverse characteristics [32–36,113]. First, unlike cohesin/CTCF loops (usually ~several to over ten thousand in number), which are globally lost upon cohesin deletion [32–34], E–P loops are largely insensitive to acute depletion (3 hours) of CTCF or cohesin, pointing to a loop extrusion-independent mechanism for E–P loop formation or maintenance [36]. In agreement, an independent study also observed that a subpopulation of loops, which are frequently anchored at superenhancers, was not affected by cohesin deletion [34]. Second, the strength of E–P loops is positively correlated with the gene expression levels, supporting an involvement of transcriptional regulation; meanwhile, cohesin loops do not show such correlation [36]. Furthermore, overall strength of cohesin/CTCF loops is stronger than that of E–P loops [36], but acute disruption of the former only caused very mild transcriptomic changes, indicating that cohesin/CTCF loops serve more as ‘structural loops’ [34,36]. Also, note that, compared to acute deletion, long-term loss of cohesin/CTCF has a far more dramatic effect on the transcriptome, with hundreds to thousands of genes showing expression change [32,33,36,98,113,115]. Thus, E–P loops may only temporarily sustain the gene expression program in the absence of structural loops [36]. In agreement with this notion, numerous studies have previously demonstrated that genome editing of specific CTCF-bound TAD boundaries, or manipulation of specific cohesin/CTCF loops, can significantly impact E–P contacts and expression of the nearby genes [116–118].

The presence of IDPs is a feature common to all organisms (including bacteria, archaea, and eukarya), and the abundance of disordered protein sequences increases proportionally with complexity of the organism, with approximately 52–67% of eukaryotic proteins carrying IDRs longer than 30 amino acids [61,119]. Unlike a ‘lock-and-key’ model, which explains interaction of highly structured protein domains, IDRs rely on weak, multivalent interactions to form phase-separated condensates [58–62]. Many TFs and (co)regulators harbor IDRs and undergo LLPS [58–62]. Next, we will discuss about current understanding as of how the LLPS ability of gene/chromatin-regulatory factors contributes to chromatin looping.

### (1) IDR-containing oncoproteins such as NUP98-HOXA9, EWS-FLI, and ER-alpha

IDPs/IDRs are frequently involved in cancer-associated mutations such as aberrant fusion, suggesting a common oncogenic mechanism [23,63]. For example, NUP98-HOXA9 (Figure 1b), a chimera generated by fusion between the DNA-binding domain of HOXA9 (a homeodomain-containing TF) and the FG-repeats-containing segment of NUP98, is associated with development of leukemias. The FG-repeats sequence, or IDR, of NUP98 has a potent capability to induce phase separation *in vitro* [82,83,120–122]. In cells, the NUP98-HOXA9 oncoproteins also display various features highly consistent with LLPS [82,121,122]. Similar LLPS characteristics were found with other leukemia-associated NUP98 onco-fusions such as NUP98-PRRX1, NUP98-KDM5A, and NUP98-NSD1 [121–123]. By mutating the IDR of NUP98-HOXA9 (Phe-Gly repeats) to a LLPS-incompetent sequence (Ser-Gly repeats) or by decreasing the FG repeat valency, Ahn et al. found that the LLPS-forming capability of NUP98-HOXA9 is essential for establishing the super-enhancer-like binding pattern seen with this onco-TF, is required for promoting CTCF-independent E-P looping among cognate sites bound by NUP98-HOXA9 (such as cis-regulatory elements of the *PBX3, HOXA* and *HOXB* proto-oncogenes), and is also required for proto-oncogene activation and leukemogenesis [82]. An artificial chimera FUS-HOXA9, which was created by replacing the NUP98 IDR with a LLPS-competent IDR of FUS (a Tyr-Ser-rich sequence [81]), largely recapitulated the genome-regulatory effects of NUP98-HOXA9, such as super-enhancer-like binding, proto-oncogene activation, and leukemic induction [82].

Such a role of TF LLPS in enhancing loop formation can potentially be generalized to other cancer-related chimeras that display features similar to NUP98-HOXA9 [82] (Figure 1b). Indeed, EWS-FLI, a hallmark fusion of Ewing’s sarcoma (a very aggressive pediatric bone cancer), also contains a LLPS-inducing IDR from EWS (a Try-Ser-rich sequence [81]) and a DNA-binding domain from FLI, an ETS family TF. Bouley et al. showed that the LLPS-inducing IDR of EWS confers a phase-transition property to EWS-FLI, leading to genomic retargeting of EWS-FLI-associated chromatin-remodeling complex (i.e. the BAF complex) and induction of an oncogenic gene-expression program [124]; also, fusing a minimal LLPS-inducing IDR sequence to FLI was sufficient to recapitulate the EWS-FLI-related activities [124]. A more recent study further reported that EWS-FLI induces looping [125], reminiscent of what was observed with NUP98-HOXA9 [82]. Specifically, new DNA loops are formed with their anchors associated with binding of EWS/FLI and not CTCF [125]; in addition, EWS/FLI binds to the GGAA-rich motifs in the genome of Ewing’s sarcoma, leading to new enhancer formation, A-type compartmentalization, and target gene upregulation [125].

LLPS of IDP can significantly influence the compartmentalization and biological function of interacting partners, as previously proposed in a scaffold–client model [65,126]. In this model, LLPS of the scaffold protein establishes the structure of condensates, whereas other components, referred to as clients, are passively recruited into condensates. In such multi-component condensates (including scaffold and client), a quite diverse set of protein–protein, protein–DNA, protein–RNA, and RNA–RNA interactions can be established, leading to a coordinated regulation of enhancer/promoter activation, chromatin remodeling and looping, and gene transcription. For example, Nair et al. showed that acute 17β-estradiol-dependent activation of enhancers in the MCF7 breast cancer cells is featured with the assembly of an enhancer RNA (eRNA)–dependent ribonucleoprotein (eRNP) complex that displays properties of phase-separated condensates; such condensates are composed of a so-called ‘MegaTrans’ complex formed by a set of TFs and coactivators (such as ER-alpha, FOXA1, GATA3, p300, and mediator) [127], eRNA, and condensin [128]. Concurrent with acute stimulation of enhancers, spatial proximity of these enhancers was observed [128]. IDRs exist in many ‘MegaTrans’ components such as ER-alpha and GATA3, which indeed form liquid droplets *in vivo* and in cells [128]. A previous study has also reported co-mixing and condensation of ER-alpha and the IDR of MED1, a mediator component of ‘MegaTrans’ [129]. Based on these observations, a model was proposed that biomolecular condensates of TFs and coactivators promote formation of ‘mega-loops’ among target gene enhancers [128]. Furthermore, BRD4-NUT, the midline carcinoma-associated oncoprotein, also establishes sizable nuclear puncta or condensates, together with coactivators (such as p300) and acetylated chromatin [91–94], in a manner consistent with the scaffold–client model; in addition, BRD4-NUT condensates appear to serve the hub for promoting long-range intra-chromosomal and inter-chromosomal interactions among regions targeted by BRD4-NUT [95]. It merits more thorough investigation whether and how a broader range of oncoproteins use IDR/LLPS-driven mechanisms to orchestrate the re-distribution of gene/chromatin-regulatory factors, generation of aberrant long-range chromatin loops/contacts, and deregulation of target gene expression, leading to oncogenesis.

### (2) IDR-containing tumor suppressors UTX and UTY

In addition to oncoproteins, IDR and phase separation also regulate the function of tumor suppressors. Recently, Shi et al. reported that an IDR within UTX (also known as KDM6A) forms phase-separated liquid condensates [130]. UTX acts as a H3K27-specific demethylase, and interestingly, loss-of-function mutation at the UTX IDR is recurrent in human cancers, indicating a role in tumor suppression [130]. By employing a set of IDR deletion, mutagenesis, and replacement strategies, the researchers elegantly demonstrated a critical role for UTX IDR and phase separation in potentiating its chromatin modulation and tumor suppression functions [130]. Besides the intrinsic H3K27 demethylase activity, UTX can additionally recruit the H3K4 methyltransferase, MLL4 (also known as KMT2D), into the same condensates where the UTX-MLL4 complex removes H3K27me3 and induces H3K4 methylation. Moreover, high-order chromatin interactions were established by UTX, a process dependent on its IDR and condensation-forming property – here, Hi-C followed by chromatin immunoprecipitation (HiChIP) for H3K4me3 and H3K27ac detected that loss-of-function mutation of the UTX IDR caused a partial disruption of those long-range E–P looping patterns normally seen in WT cells, which was also correlated with changes in gene expression [130].

UTX represents one of a few tumor suppressors known to escape X inactivation and contribute substantially to a higher rate of cancer susceptibility seen with the male than the female [131]. The homolog of UTX on the Y chromosome is UTY (also known as KDM6C). Relative to UTX, male-specific UTY exhibited a weaker tumor-suppressive activity in the leukemia models [130]. While deletion of the UTY IDR reduced its tumor-suppressive activity, replacing it with the IDR of UTX significantly enhanced UTY’s activity; conversely, replacing the IDR of UTX with that of UTY significantly reduced UTX’s tumor-suppressive activity, pointing out an important role for IDR [130]. Interestingly, the UTY IDR actually exhibits a stronger phase-separation capability than the UTX IDR, consistent with a fact that the former contains more aromatic residues and more abundant blocks of oppositely charged residues [130]. Compared with condensates of the UTX IDR, those of the UTY IDR were less liquid-like, displayed the slower dynamics, and adopted a more solid-like material state [130]. In addition, certain cancer-associated somatic mutations target the UTX IDR and condensates of such UTX-IDR mutants were more ‘hardened’ and less fluid than WT controls [130]. In breast cancer cells, evidence also exists to show that the ‘MegaTrans’ complex can progressively transition from a fluid to a more viscous solid state, in response to long-term treatment of ER-alpha ligand [129]. Thus, a balanced condensation ability and appropriate material state (such as a metastable liquid state versus a solid/gel-like state) can regulate physiological activities of gene/chromatin regulators.

### (3) The pluripotency factor OCT4

It is generally thought that TADs are stable across diverse cell types and conserved across species [3]; however, TAD reorganization occurs during the transition of cellular states, such as somatic cell reprogramming [132]. Induced reprogramming of mouse embryonic fibroblasts (MEFs) to pluripotent stem cells (PSCs) is associated with significant changes in TAD boundaries and size, leading to TAD shift, fusion, or separation, which is correlated with changes in gene expression and cellular identity [132]. For example, merging of the TAD containing Dppa5a, a pluripotency gene, with a neighboring TAD was observed during reprogramming [132]. In order to dissect the role for TAD reorganization in reprogramming, Wang et al. used a dCas9-based chemical-inducible linking approach to artificially fuse the above two TADs, which are separated by a CTCF-bound insulator in MEFs [132]. Such induced merging of TADs led to decreased CTCF binding at the insulator and early activation of Dppa5a expression during reprogramming; compared to controls, these engineered MEFs also showed enhanced efficiency of reprogramming [132]. These effects can be recapitulated by deletion of the CTCF-bound insulator in MEFs [132]. To further dissect the mechanism underlying TAD reorganization, the authors turned to OCT4, a critical reprogramming TF, and mapped OCT4-associated loops by HiChIP [132]. OCT4 loop clusters (including both E-P and P-P loops) showed a positive correlation with TAD reorganization – there was an overall increase of OCT4 loops at TAD boundary sites that disappeared during cell transitioning to PSCs; conversely, there was an overall decrease of OCT4 loops at those boundary sites newly formed in PSCs [132]. In both boundary groups, either lost or newly formed in PSCs compared to a pre-PSC state, overall CTCF binding was enhanced upon acute depletion of OCT4 in PSCs, suggesting antagonism between OCT4-associated loops and CTCF-associated structural loops [132]. This observation is somewhat reminiscent of what was seen with LLPS-competent NUP98-HOXA9, which led to generation of new loops between binding sites and concurrent loss of nearby loops anchored by CTCF (for example, see the MAP2K5 locus [82]).

OCT4 (Figure 1c) harbors LLPS-inducing IDRs [129,132]. It can phase separate *in vitro* and form nuclear droplets in PSCs [129,132]. Combined analyses of DNA fluorescence in situ hybridization (FISH) and immunofluorescence (IF) showed co-localization between OCT4 condensates and OCT4 loop cluster-targeted genomic regions [132]. Importantly, disruption of OCT4 phase separation via independent approaches (either substitution of an acidic-residue-rich IDR with alanine or a small three-amino-acid deletion within a C-terminal IDR) significantly attenuated the TAD reorganization normally observed upon ectopic expression of WT OCT4 in MEFs; also, these phase-separation-defective mutations attenuated the efficiency of reprogramming by OCT4 [132]. Furthermore, fusing the phase-separation-incompetent OCT4 with an independent LLPS-inducing IDR from FUS restored OCT4 LLPS and also largely restored the TAD reorganization and reprogramming capabilities [132].

### (4) Polycomb proteins

Polycomb repressive complexes 1 and 2 (PRC1 and PRC2) represent a group of gene silencing-associated chromatin modifiers [133–135]. There exist canonical and noncanonical PRC1 and PRC2, which differ in complex composition and biological function; for the details, readers shall refer to other reviews [133–135]. PRC1 and PRC2 can form condensates [136–144]. In particular, IDRs harbored within CBX2 and PHC1, the two subunits of canonical PRC1, were reported to drive LLPS *in vitro*; in animal models, compaction of PRC1 target chromatin also exhibited an IDR dependency [136–144]. Evidence based on Hi-C and other mapping methods further showed the existence of polycomb-associated loops including P–P and E–P interactions [36,145–150]. PRC1/2 is also involved in the formation and/or maintenance of so-called polycomb-associated domains (PADs), which refer to compact, self-associating domains of polycomb-targeted chromatin [145,146,151–154]. Polycomb-associated long-range E–P interaction or PAD formation was often found to be dependent on PRC1, and not H3K27me3 [153,155] or CTCF [154]. Thus, LLPS of PRCs is potentially involved in the regulation of the 3D chromatin structure, but further investigation is merited to dissect the molecular detail.

## Concluding remarks

As discussed above, emerging evidence shows that IDR-containing gene/chromatin-regulatory factors form condensates and are involved in generation of new chromatin loops, a process dependent on IDR and its LLPS-forming ability (as seen with NUP98-HOXA9 [82] and UTX [130]). Additionally, OCT4-associated loops were shown to have a significant impact on reorganization of higher-order 3D structures such as TADs, and this process also relies on the IDR- and LLPS-inducing capability of OCT4 [132]. Numerous TFs (such as cMyc and ER-alpha [129,156]) and coactivators (many of which are chromatin modifiers, remodelers, or readers such as p300 [157], ENL [158], and BRD4 [159,160]), as well as mediator [160] and RNA polymerase II [103,161–163], all contain IDRs that can induce LLPS *in vitro* and mediate condensate formation *in vivo*. Furthermore, classic heterochromatic factors, such as HP1-alpha [84,85], PRC1/2 [138,139,141], and MeCP2 [164–166], also contain IDRs and undergo LLPS. Thus, it is tempting to speculate that the contribution of phase separation and IDPs to the 3D chromatin structure (re)organization is a widespread phenomenon occurring at all chromatin compartments/domains and across different genomic scales [50,55–57], which awaits additional studies.

Conceivably, the IDP-/LLPS-associated chromatin loops and the CTCF-/cohesin-formed structural TADs/loops operate independently and can cross-talk as well, in either a cooperative or antagonistic manner. How exactly these two 3D structure classes influence one another seems rather complex. On the one hand, it has been long postulated that CTCF-/cohesin-mediated structural TADs/loops can constrain the communication between a E–P pair separated by CTCF, which has gained much support from genetic manipulation studies of specific CTCF sites/loops [116,117]; meanwhile, CTCF binding sites, either within TADs or close to promoters, can also stabilize or directly promote E–P and P–P contacts, potentially contributing to gene activation [113,115]. In support of the latter notion, a recent work further showed that CTCF-mediated structural loops provide a topological framework for the formation of transcriptional condensates by TFs, coactivators, and RNA polymerase II [167]. However, acute disruption of CTCF/cohesin does not significantly affect gene expression at a global level [32–34] nor does it immediately affect a vast majority of E–P and P–P loops, leading to postulation of a ‘time-buffering’ model [36]. Also, about 20% of E–P loops and one third of P–P loops can go across TAD boundaries and still retain a comparable level of contact intensity to those equidistant loops located within the same TADs, which then seems to argue against the constraint model as to E-P communication [36]. On the other hand, IDP-/LLPS-associated loops can have a local effect on structural loops. For instance, loop clusters associated with phase-separated OCT4 seem to antagonize CTCF binding and local TADs during the somatic cell reprogramming model [132]. Another example is that ectopic expression of LLPS-competent NUP98-HOXA9 promotes new looping between its binding sites at the MAP2K5 locus, concurrent with loss of a nearby CTCF loop [82]. Furthermore, lower-order 3D structures such as OCT4-associated loops can have a significant impact on higher-order structures such as TADs [132]. Whether such effects can be generalized to other IDPs remains unclear, and additional studies are warranted to specify rather complex crosstalk between structural loops and E–P loops.

Finally, it is worth mentioning that some previous studies of phase separation relied heavily on non-physiological over-expression and artificial systems, which represents a concern [65,168,169]. New tools for more definitely determining phase separation *in vivo* need to be developed. Also, how exactly the E–P contact/looping regulates gene expression remains to be more clearly determined in the future [114]. For instance, a live cell imaging-based study of Sox2 in ESCs showed that stable and direct pairing of the Sox2 promoter with enhancer is unlikely to explain transcriptional activation of Sox2 in real time [170]. Furthermore, olfactory receptor genes are known to establish long-range interactions (including inter-chromosomal ones) and form a multi-chromosomal hub so that all but one olfactory receptor gene is repressed in olfactory sensory neurons [171]; however, the underlying mechanism remains elusive. All the aforementioned outstanding questions await further investigation.
